# Clinico-radiological characteristics and lethality of HIV-tuberculosis coinfection in the Infectiology ward of the Libreville University Hospital, Gabon

**DOI:** 10.4102/sajid.v40i1.695

**Published:** 2025-04-24

**Authors:** Michele Marion Ntsame Owono, Charleine Manomba Boulingui, Magalie Essomeyo Ngue Mebale, Marielle Karine Bouyou Akotet

**Affiliations:** 1 Department of Infectiology, University Hospital, Libreville, Gabon; 2 Department of Medicine and Medical Specialties, Université des Sciences de la Santé, Libreville, Gabon; 3 Centre de Recherche en Pathogènes Infectieux et Pathologies Associées, CREIPA, Université des Sciences de la Santé, Libreville, Gabon; 4 Department of Basic Sciences, Faculty of Medicine, Université des Sciences de la Santé, Libreville, Gabon

**Keywords:** HIV–tuberculosis, coinfection, mortality, Gabon, CD4 cells, ART

## Abstract

**Background:**

HIV advance disease and tuberculosis (TB) are still frequent in Gabon.

**Objectives:**

This study described the clinical and radiological features of bacteriologically confirmed TB among hospitalised persons living with HIV (PLHIV) and in-hospital death-associated factors.

**Methods:**

Patients older than 18 years old, with a diagnosis of TB between 2021 and 2022, were prospectively included. Sociodemographic, clinical, radiological data, CD4 cell count, ART, lenght of hospital stay and mortality were recorded and analyzed. Factors associated with patient death were investigated.

**Results:**

Overall, 94 (54.7%) of 172 hospitalised PLHIV had TB. Their median age was 37 (32–42) years, 67.0% were females, 47.9% were on ART and 85.0% were in the advanced disease stage. Overall, 52 (55.3%) PLHIV had isolated pulmonary TB, 13 (13.8%) had extra-pulmonary forms, mainly neuromeningeal and lymph node forms, 25(26.6%) had a disseminated TB that involved pulmonary lesions and 4 (4.3%) had an extra-pulmonary disseminated TB. The median CD4 count was 83 (54–128) cells/µL. It was lower in the group of deceased participants (*p* = 0.04). The case fatality rate was 26.0% (*n* = 24). Mortality associated factors were length of hospital stay below 10 days (odds ratio [OR] = 3.9 [1.06–14.3], *p* = 0.04) and CD4 < 200 cells/mm^3^ (*p* = 0.01). A trend was also observed for males (OR = 2.11 [0.81–5.5], *p* = 0.062) and age above 45 years (OR = 2.68 [0.92–7.78], *p* = 0.07).

**Conclusion:**

HIV–TB coinfection and extra-pulmonary forms are still frequent in immunocompromised PLHIV. The in-hospital mortality is high, probably because of late diagnosis.

**Contribution:**

This study highlights the need of integrated early HIV and TB diagnosis and management in highly endemic settings to improve coinfected patient outcome.

## Introduction

Tuberculosis (TB) still remains a public health problem, particularly in developing countries, where more cases have been observed over these last 5 years. In 2023, the World Health Organization (WHO) reported 10.6 million cases, of whom 7.5 million were new cases; 1.13 million died, including 167 000 people living with HIV (PLHIV).^1^ HIV infection increases the risk of developing TB, each disease worsening the prognosis of the other.^2^ HIV is a risk factor for the reactivation of latent *Mycobacterium tuberculosis* infection resulting in TB disease, whereas *M. tuberculosis* accelerates the natural progression of HIV to AIDS.^2,3^ The negative impact of HIV on the course of TB is also frequently described in sub-Saharan Africa.^4^

Tuberculosis is the main fatal opportunistic disease among people living with HIV in some African settings.^5,6^ The lethality of HIV–TB co-infection is high, as is the risk of relapse in survivors.^1,2,3,4^ Moreover, the clinical and radiological pictures of TB can be polymorphous and non-specific in this specific population, delaying diagnosis and leading to delayed management.^7,8^ Some studies reveal a predominance of extra-pulmonary and disseminated forms in immunocompromised patients, while others report a predominance of pulmonary forms.^9,10^

In Gabon, the incidence of TB was 509 cases per 100 000 inhabitants in 2023, according to WHO.^11^ In the main cities of its nine provinces, HIV diagnosis and care, TB diagnosis and management are easily accessible to the population through dedicated treatment centres and laboratory facilities. At the national level, all TB-diagnosed patients receive free case management. However, in very remote areas, equipment for laboratory TB diagnosis and, to a lesser extent, medical radiology are often lacking. Tuberculosis diagnosis and management are based on clinical symptoms and patient medical history.

The development of contextualised decision-making algorithms that consider the risk factors and patient profile facilitates identifying patients at risk of death from others.

So far, the clinical epidemiology of TB and factors associated with pulmonary or extra-pulmonary TB are scarcely described as are case fatality rate and factors associated with death during HIV–TB coinfection.

The aim of this study was to determine the clinical and radiological profile and the lethality of TB–HIV coinfection in patients hospitalised in the infectious Diseases Department of Libreville, the capital city of Gabon.

## Research methods and design

### Study setting

This study was conducted at the Infectious Disease ward of the main public hospital of Gabon, the Centre Hospitalier Universitaire de Libreville (CHUL), the national reference centre for HIV care. It is in Libreville, the capital city of Gabon, where 60% of the total population, including most PLHIV, live. Patients are referred from other health structures including the peripheral ambulatory treatment centres for infectious disease management. It is the single inpatient ward specialised for infectious disease management as well as HIV-advanced disease and opportunistic infection treatment and follow-up. Each year, almost 250–300 PLHIV are hospitalised in this ward.

### Study design

This was a prospective descriptive and analytical study conducted during over 1 year, from October 2022 to October 2023 in the CHUL Infectious Diseases Department.

### Study population

The study population consisted of PLHIV hospitalised for a bacteriologically confirmed TB during the study period. Participants were included if they had the following criteria: age ≥ 18 years old, laboratory confirmed diagnosis of *M. tuberculosis* (according to WHO bacteriologically confirmed TB case definition), availability of medical records including results of medical imaging, culture and histopathology and acceptance to participate in the study by providing a written informed consent.

### Study variables

The following data were collected on a case report form: age, gender, marital status, occupation, history of TB, contact with an active TB patient, time since HIV diagnosis, antiretroviral treatment (ART), ART duration, comorbidities, CD4 cell count, fever or history of fever, clinical signs, the anatomical site(s) of TB (clinical form), length of hospital stay and hospitalisation outcome.

Some laboratory test results were also recorded, namely: haemoglobin level, urea, creatinine and liver function tests.

### Tuberculosis diagnosis and anatomical classification

According to WHO recommendations, active pulmonary or extra-pulmonary TB was suspected on the basis of a combination of the following: (1) medical history and clinical evidence including WHO four-symptoms screen (W4SS) that comprises anyone with current cough, fever, night sweat and weight loss^12^ and (2) radiological and/or histopathological and/or positive culture evidence, depending on patient clinical symptoms. All these additional non-clinical tests were used for the TB anatomical site determination and included chest X-ray, CT scan, histopathology, smear microscopic examination or culture.

For all the patients, the bacteriological confirmation of *M. tuberculosis* was based on the molecular detection of *M. tuberculosis* by real-time nested Polymerase Chain Reaction (PCR) assay using the GeneXpert MTB/RIF, as well as the rifampicin resistance in either clinical samples or culture isolates, according to standard procedures. Indeed, culture was rarely performed because as per national recommendation and routine procedures of the infectiology ward, the GeneXpert analysis should be performed without any additional cost for all confirmation and notification of TB cases. Histopathology, CT scan, chest X-ray, bone scan, smear and culture were performed when appropriate.

Sputum was analysed in case of suspected pulmonary TB; lymph node aspirates, pleural and pericardial fluids, gastric aspirates, urine and cerebrospinal fluid were collected and analysed for suspected extra-pulmonary TB.

For the extra-pulmonary forms, culture, histopathological analysis of biopsies, bone scan, CT scan and chest X-ray were performed together with the *M. tuberculosis* molecular detection when appropriate.

Bacteriologically confirmed pulmonary TB was defined when only lungs were compromised, according to X-ray or CT scan and GeneXpert results.

Extra-pulmonary forms were those with bacteriologically confirmed TB affecting any other system or organ except the lungs, according to CT scan, bone scan CT, X-ray, histopathology or echocardiography.

Disseminated TB was defined when at least two different organs or systems were involved.

### Statistical analysis

Data were entered and analysed using Statview 5.0. Qualitative data were analysed using the chi-square or Fischer exact test, while quantitative ones were analysed using the Mann-Whitney and Kruskal-Wallis tests. Crude odds ratio (OR) with 95% confidence interval (95% CI) was used to assess the association between the studied variables and patient death. A *p*-value ˂ 0.05 was considered significant.

### Ethical considerations

Patients were included voluntarily after obtaining their written consent for the use of their data. The study was approved by the National Ethics Committee for Research (CNER) (reference no.: 026/2022/CNE/SG/P). It was authorised by the Director of Medical Affairs and the Head of the Department of Medicine and Medical of the CHUL. Each patient had an identification number used for data analysis.

## Results

### Patient characteristics

During the study period, out of 172 hospitalised PLHIV, 94 had confirmed TB, representing a hospital prevalence of 54.7%. Women comprised 67% of the cohort. Most patients were single (83.0%), unemployed (52.1%) and aged between 30 and 59 years old (83.0%). (Table 1). Their median age was 37.5 (32-42) years. A total of 49 (52.1%) were not on ART at TB diagnosis; for those already under ART on admission, the average duration of ART was 2.8 (±0.9) years. The median duration since HIV diagnosis was 3 (3-4) years, and for 6 (6.4%) patients, it was over 4 years; for 18 (19.1%), it was less than 3 years. Overall, 26 out of the 94 participants had a history of TB, while for 16 (17.0%), TB occurred in a context of the immune reconstitution inflammatory syndrome (IRIS) (Table 1).

**TABLE 1:** Sociodemographic characteristics and medical history of the 94 HIV–TB coinfected patients.

Variables	*n*	%
**Age (years)**		
< 30	11	11.7
30–59	78	83.0
≥ 60	5	5.3
**Gender**		
Female	63	67.0
Male	31	33.0
**Marital status**		
Single	78	83.0
Married	14	14.9
Widowed	2	2.1
**Occupation**		
Middle management	16	17.0
Senior executive	7	7.5
Pupil and student	5	5.3
Informal	4	4.3
Worker	13	13.8
Unemployed	49	52.1
Previous history of tuberculosis	26	27.7
Close contact with an active TB patient	3	3.2
**ART**		
On ART	45	47.9
ART naïve	40	42.6
ART discontinued	9	9.6
**TB IRIS**	16	17.0
Unmasking	7	7.4
Paradoxical	9	9.6

TB, tuberculosis; ART, antiretroviral therapy; IRIS, immune reconstitution inflammatory syndrome.

Other opportunistic infections were diagnosed in seven (7.4%) patients: six had a cerebral toxoplasmosis and one had a progressive multifocal leukoencephalopathy. Their cerebrospinal culture was not positive for cryptococcus.

### Clinical symptoms

At least two of the WHO four symptoms included in the WHO-recommended four symptom screen (W4SS) were found in all the participants, and all were common (Table 2); all reported weight loss, 89.4% reported cough, 85.1% reported night sweats and 68.1% had fever on admission. Other signs were dyspnoea, non-pregnant amenorrhoea and haemoptysis. A total of 77 (81.9%) PLHIV had pulmonary lesions, neuromeningal and lymph node forms predominated among the 17 with extra-pulmonary TB (Table 2). More precisely, only 52 (55.3%) out of the 77 with pulmonary lesions had isolated pulmonary TB. The main radiological abnormalities observed were a consolidation, the presence of infiltrates, the pleural effusion, lymph node enlargement and a miliary pattern (Table 2). Non-disseminated extra-pulmonary TB was diagnosed in 13 (13.8%) participants; disseminated forms were found in 29 (30.9%), of whom 25 (86.2%) had associated pulmonary involvement and four a disseminated TB without lung lesions.

**TABLE 2:** Clinical and paraclinical characteristics of the 94 HIV–TB coinfected patients.

Characteristics	*n*	%
**Clinical signs** ^†^		
Fever	64	68.1
Cough	84	89.4
Weight loss	94	100.0
Night sweats	80	85.1
Loss of appetite Dyspnoea	49 24	52.1 25.5
Non-gravidic amenorrhea	24	25.5
Ascitis	12	12.7
Haemoptysis	2	2.1
**Extra-pulmonary forms** ^†^		
Neuromeningeal	5	29.4
Bone	3	17.6
Pericarditis	1	5.8
Pleural	2	11.7
Peritoneal	4	23.5
Lymph nodes enlargement	13	13.8
**Chest radiological and CT scan findings** ^†^		
Consolidation	21	22.3
Cavitation	8	8.5
Lymph nodes	12	12.7
Interstitial	25	26.5
Miliary	13	13.7
Pleural effusion	13	13.8
Nodules	14	14.8
Destruction of vertebral bodies	3	3.2
Paravertebral abscess	2	2.1
Calcifications	3	3.2
**CD4 cell count cells/mm** ^**3**^ **)**		
< 50	23	24.4
50–99	37	39.3
100–199	20	21.2
≥ 200	14	15.0

CT, computed tomography.

† , Some patients could have multiple features or lesions.

### Biological abnormalities

CD4 cell counts ranged from 3 cells/mm^3^ to 330 cells/mm^3^, with a median of 83 (54–128) cells/mm^3^; 80 (85.1%) participants were immunosuppressed, among them 60 (63.4%) had less than 100 CD4 cells/mm^3^ (Table 2). The median CD4 cell count was lower in those on ART (75 [44–117] cells/mm^3^) compared to the naïve ones (87 [66–170] cells/mm^3^) or to the nine who had discontinued ART (88 [69–170] cells/mm^3^) (*p* = 0.047). In fact, 87.3% of PLHIV on ART, 58.3% of the naïve ones and five out of the nine who discontinued ART had less than 100 CD4 cells/mm^3^ (*p* = 0.49). The median CD4 cell count was significantly lower in case of extra-pulmonary TB (67 [24–84 cells/mm^3^]) than in pulmonary forms (89 [56–138 cells/mm^3^]) (*p* = 0.01). There was a trend towards a lower median CD4 cell count in the group or patients who were on ART under 3 years (56 [34–103] cells/mm^3^) compared to those with longer treatment duration (87 [63–138] cells/mm^3^) (*p* = 0.08). Moreover, PLHIV with a short hospital stay had a lower median CD4 cell count (76 [26–100] cells/mm^3^) compared to those with more than 10 days hospital stay (95 [66–170] cells/mm^3^) (*p* = 0.01).

The median haemoglobin level was 9.5 (7.9–10.7) g/dL; 76 (80.8%) of study participants were anaemic, out of whom 42 had moderate anaemia (5.1 g/dL – 8.9 g/dL). High liver function enzyme levels and impaired renal function were observed in five (5.3%) and 14 (15%) PLHIV, respectively. Two people (2.1%) had Rifampicin-resistant TB.

### Mortality and associated factors

**FIGURE 1:**
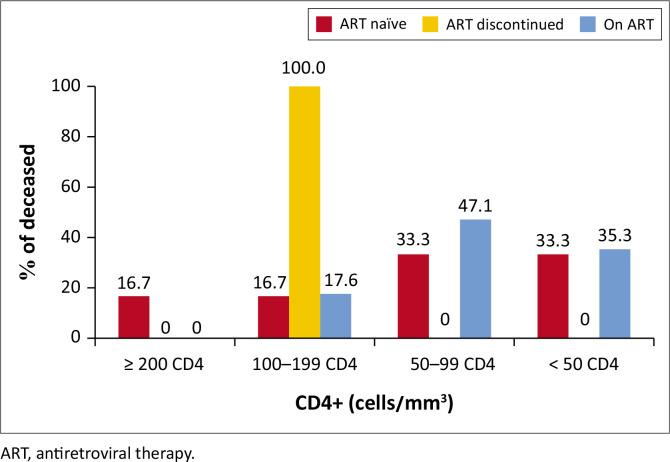
Frequency of deceased PLHIV stratified by antiretroviral therapy uptake and CD4 count.

Case fatality rate was 26% (*n* = 24/94). In 87.5% of cases, death occurred before the 10th day of hospitalisation. Indeed, the median length of hospital stay was 7.0 [6.0–8.0] days in those who died and 12.0 (10.0–14.0) days in those who survived (*p* < 0.01). The median age was 40 (33–47) years and 37 (32–41) years in these two groups, respectively (*p* = 0.19). Patients aged more than 45 years tended to be at higher risk of death compared to the youngest (*p* = 0.07). The occurrence of death was neither related to the time since HIV diagnosis, nor to a recent history of TB (Table 2). Death was significantly more frequent in case of immunosuppression (28.8% in the group with CD4 < 200 cells/mm^3^ vs. 7.1% in the group above 200 CD4 cells/mm^3^; *p* < 0.01). This trend was also observed according to ART, none of the PLHIV under ART who died had more than 200 CD4 cells/mm^3^, 82.4% (*n* = 14/17) of them had less than 100 CD4 cells/mm^3^ (Figure 1).

The median CD4 count was lower in the group of patients who died (76.0 [38–100] CD4 cells/mm^3^) than in those who survived (89 [56–150] CD4 cells/mm^3^) (*p* = 0.04). There was a trend towards higher case fatality in males and in cases of extra-pulmonary involvement (Table 3).

**TABLE 3:** Factors associated with inpatient death in patients with HIV–TB coinfection (*N* = 24).

Factors	*n*	%	OR	95% CI	*p*
**Gender**					
Female	13	20.6	1.00	-	-
Male	11	35.5	2.11	0.81–5.5	0.062
**Age (years)**					
< 45	16	20.8	1.00	-	-
≥ 45	8	42.1	2.68	0.92–7.78	0.070
**Time since HIV diagnosis**					
≤ 3 years	12	22.6	1.00	-	-
> 3 years	12	29.3	1.41	0.56–3.59	0.470
**On ART**					
ART naïve	6	15.0	0.33	0.12–0.90	0.030
ART discontinued	1	11.1	0.21	0.03–2.04	0.190
On ART	17	37.8	1.00	-	-
**Current ART duration†**					
ART ≤ 2 years	7	41.2	1.50	0.54–5.8	0.350
ART > 2 years	10	31.3	1.00	-	-
**Recent history of tuberculosis**					
Yes	8	30.8	1.44	0.53–3.94	0.470
No	16	23.5	1.00	-	-
**Length of hospital stay**					
< 10 days	21	87.5	155.00	29.3–833.3	0.010
≥ 10 days	3	4.3	1.00	-	-
**CD4 count (mm** ^**3**^ **)**					
< 50	8	34.8	6.93	0.76–63.0	0.085
50–99	10	27.0	4.81	0.56–41.7	0.150
100–199	5	25.0	4.33	0.45–42.02	0.210
≥ 200	1	7.1	1.00	-	-
**Clinical forms of tuberculosis**					
Isolated pulmonary	13	25.0	1.00	-	-
Lung + other localisations	5	20.0	0.75	0.23–2.4	0.620
Extra-pulmonary	6	35.3	1.64	0.50–5.30	0.420
**Other opportunistic infection**					
Yes	3	42.3	2.35	0.49–11.33	0.290
No	21	23.1	1.00	-	-

OR, odds ratio; CI, confidence interval; ART, antiretroviral therapy.

† , PLHIV who discontinued ART not included.

## Discussion

Despite the decrease in HIV prevalence in Gabon and the efforts for a better access to TB diagnosis and treatment, the TB burden is still high.^13^ This prospective observational study described TB’s sociodemographic, clinical patterns in PLHIV hospitalised in the main inpatient ward of Gabon. As previously reported, this study revealed a high hospital HIV–TB coinfection prevalence (54.7%). *Mycobacterium tuberculosis* is the most frequent opportunistic infection and the most common reason for HIV diagnosis in Gabon.^14,15,16^ In neighbouring Cameroon, this rate was 27.9%.^6^ The fact that the CHUL is the referral hospital for infectious diseases inpatient management could also partly explain the high TB rate in hospitalised PLHIV.

Tuberculosis incidence and new cases increased worldwide since 2020, reaching 8.2 million in 2023 from 5.8 million in 2020. The highest burden of TB–HIV coinfection still remains in the WHO African Region.^17^ Similar to reports from several countries from sub-Saharan Africa, late diagnosis and late entry into care would explain this high frequency of HIV–TB coinfection, among PLHIV inpatients in the present study.^18,19^ In a survey performed in two ambulatory HIV clinics in Libreville, 46% of PLHIV had delayed entry into care, while 45% had advanced HIV disease at the time of entry into care.^16^ Among the 94 study participants who knew their HIV status since more than 2 years, 49 were still not on ART, more than 8 years after the Test and Treat adoption in the country. Even in some settings outside sub-Saharan Africa, the proportion of PLHIV who do not seek care after the HIV diagnosis can reach more than 80%, AIDS-defining illness is often the main reason for HIV testing and diagnosis.^20,21^

Women represented two thirds (67.0%) of the HIV–TB coinfected patients. This female predominance has been reported by other authors, whereas in Guatemala and Colombia, males represented the majority of coinfected patients.^7,8,9,21,22,23,24^ Moreover, women represent more than 60% of the PLHIV involved in the continuum of care in Gabon; they seek medical advice more often than men.^25^

The mean age of HIV–TB coinfected patients was 39.3 years old in the study population and 38 years in Enugu State in Nigeria.^26^ A similar trend was recorded in Cameroon between 2019 and 2021.^27^ According to the literature, TB and HIV infection preferentially affect sexually active adults within the 35–45 years age bracket.^6,7,28^

Tuberculosis affects all social strata. Unemployed people accounted for 52.1%, whereas the proportion of senior executives was low (7.5%). This confirms that in Libreville like in other countries, TB is linked to poverty.^29,30^

Clinically, cough (89.4%), fever (68.1%), sweating (85.1%), dyspnoea (25.5%) and weight loss (100%) were the main signs found as reported elsewhere.^6,23,31,32^ Four of these five symptoms are used for the screening of TB in suspected PLHIV according to the WHO W4SS.^33^ It was already reported that PLHIV more frequently presents active TB and symptomatic TB, generally in more than 70% of cases.^33^ The onset of symptoms was not recorded. The study participants do not seem to have a correct medical follow-up, so they probably consulted very late when they had several symptoms. Furthermore, this present survey was conducted during the coronavirus disease 2019 (COVID-19) period, and another respiratory viral coinfection cannot be excluded in the absence of a specific screening.

The main site of the infection was the lung, with or without extra-pulmonary forms involving neuromeningeal (29.4%), peritoneal (23.5%) or lymph nodes (13.8%). However, extra-pulmonary forms were the most frequently reported by Sylla et al. in Cote d’Ivoire, whereas pulmonary and lymph node forms predominated in Thailand.^32,34^

At the time of TB diagnosis, almost 50% of the patients were on ART, the majority of them since more than 18 months. Treatment observance and compliance were not checked. Nevertheless, Anua in Cameroon found an association between non-compliance to ART and TB.^35^ Apart from biological reasons, difficulties in treatment adherence and disengagement from the continuum of care are some social reasons that likely contribute to lack of ART effectiveness.^36^ The study participants were mostly unemployed; this status can be responsible for disengagement from ART and disengagement from the HIV clinic because of competing survival priorities. In addition, health worker attitude towards PLHIV, lack of time for discussion with patients during their visit for treatment dispensation, patient’s feeling well without symptoms and economic stress and stigma are several factors requiring consideration in ART patients with advanced HIV disease.^37,38^

In-hospital mortality was 26.0%, lower than that reported by Shimazaki et al. in the Philippines (37.5%) and Berkchi in Morocco (36%) but higher than that reported by Diatta et al. (9.5%) in Senegal.^39,40,41^

As reported in South Africa, men tended to be at higher risk of mortality.^42^ Similarly, a trend towards a higher mortality and CD4 cell count below 50 cells/mm^3^ was observed. Globally, poor ART adherence, HIV-advanced disease, low CD4 cell count, absence of cotrimoxazole and Isoniazid preventive therapies are risks recognised factors for death in HIV–TB coinfected patients.^43,44,45^ Although the univariate analysis showed a higher frequency of deaths among PLHIV aged over 45 years old (42.1%), those with extra-pulmonary TB (35.3%) and PLHIV with a concomitant opportunistic infection (42.3%), these associations were not significant in the bivariate analysis (Table 3), possibly because of the small sample size. Nevertheless, when the group of deceased patients was stratified according to the CD4 cell count, there was a predominance of low CD4 cell counts among the PLHIV on ART, which would explain why this group was at higher risk of death. Together with ART adherence and observance, a low CD4 cell count at ART initiation is also a risk factor for death.^46^ Moreover, despite ART, low CD4+ cell count and lack of TB preventive treatment remain the major risk factors of HIV–TB coinfection.^47,48^ Furthermore, HIV–TB coinfected patients face challenges in healthcare engagement for early diagnosis of TB such as difficulties in the access of TB management facilities (because of stigma, absence of knowledge and socio-economic constraints), absence of integrated HIV and TB continuum of care, difficulties in diagnosis TB in some HIV patients or absence of screening of latent TB in patients already on ART, as well as inadequate perception of healthcare providers on the importance of early TB screening and diagnosis in ART patients.^49^ Regarding the length of hospital stay, the longer hospital stay of patients who died is not surprising as they were extremely ill and died shortly after admission.

This study has some limitations. Firstly, it was a single-site study with a small sample size; the results cannot be applicable to other health structures of the country. Secondly, only PCR-confirmed cases were included; cases without *M. tuberculosis* biological detection and cases with only Urinary Lipoarabinomannan (LAM) and/or blood culture positive would have been missed, thereby leading to an underestimation of the HIV–TB burden and case fatality rate. Thirdly, this was an observational study that included participants without a complete standardisation of medical procedures. Fourthly, because of the low number of factors significantly associated with death after the bivariate analysis, and the small sample size, a multivariate analysis was not performed. To overcome this issue, deceased participant groups were categorised and compared according to their CD4 cell counts, the main variable associated with death.

Nevertheless, this is the first report of hospital HIV–TB coinfection characteristics and case fatality rate. Our study documents serious problems in both our TB and ART programmes that must be addressed. The present data could serve as basis for hospital-based and public health larger studies and for future updated HIV–TB case management recommendations.

## Conclusion

HIV–TB coinfection is common among PLHIV with advanced disease hospitalised in Libreville, with a high case fatality rate. Early diagnosis, retention into care, prompt management of HIV–TB coinfection and early introduction of preventive therapies would improve the prognosis of this coinfection
